# Enhanced Brain Disposition and Effects of Δ^9^-Tetrahydrocannabinol in P-Glycoprotein and Breast Cancer Resistance Protein Knockout Mice

**DOI:** 10.1371/journal.pone.0035937

**Published:** 2012-04-20

**Authors:** Adena S. Spiro, Alexander Wong, Aurélie A. Boucher, Jonathon C. Arnold

**Affiliations:** 1 Discipline of Pharmacology, University of Sydney, Sydney, New South Wales, Australia; 2 Brain and Mind Research Institute, Sydney, New South Wales, Australia; 3 Schizophrenia Research Institute, Darlinghurst, Sydney, Australia; Sapienza University of Rome, Italy

## Abstract

The ABC transporters P-glycoprotein (P-gp, *Abcb1*) and breast cancer resistance protein (Bcrp, *Abcg2*) regulate the CNS disposition of many drugs. The main psychoactive constituent of cannabis Δ^9^-tetrahydrocannabinol (THC) has affinity for P-gp and Bcrp, however it is unknown whether these transporters modulate the brain accumulation of THC and its functional effects on the CNS. Here we aim to show that mice devoid of *Abcb1* and *Abcg2* retain higher brain THC levels and are more sensitive to cannabinoid-induced hypothermia than wild-type (WT) mice. *Abcb1a/b* (−/−), *Abcg2* (−/−) and wild-type (WT) mice were injected with THC before brain and blood were collected and THC concentrations determined. Another cohort of mice was examined for THC-induced hypothermia by measuring rectal body temperature. Brain THC concentrations were higher in both *Abcb1a/b* (−/−) and *Abcg2* (−/−) mice than WT mice. ABC transporter knockout mice exhibited delayed elimination of THC from the brain with the effect being more prominent in *Abcg2* (−/−) mice. ABC transporter knockout mice were more sensitive to THC-induced hypothermia compared to WT mice. These results show P-gp and Bcrp prolong the brain disposition and hypothermic effects of THC and offer a novel mechanism for both genetic vulnerability to the psychoactive effects of cannabis and drug interactions between CNS therapies and cannabis.

## Introduction

Cannabis is the most widely used illicit drug in the world and may trigger psychiatric disorders such as psychosis and addiction in genetically vulnerable individuals [1]. Research identifying the exact genes that comprise genetic vulnerability to cannabis-induced brain disorders is in its infancy. A catechol-o-methyltransferase gene variant (*COMT*) increases the risk of developing schizophrenia in adolescent cannabis users [2], [3]. Our own preclinical research has shown that the schizophrenia susceptibility gene, neuregulin 1, modulates the neurobehavioural effects of the main psychoactive constituent of cannabis, Δ^9^-tetrahydrocannabinol (THC) [4], [5], [6], [7]. While studies have focused on pharmacodynamic aspects of gene-cannabinoid interactions, here we aim to explore a mechanism involving adenosine triphosphate (ATP) - binding cassette (ABC) transporters that might enhance disposition of THC in the brain.

ABC transporters are a family of drug efflux pumps that utilize ATP hydrolysis to transport substrates across biological membranes and were originally discovered due to their role in mediating multidrug resistance (MDR) to anti-cancer drugs [8]. They regulate the disposition of many drugs in tissues because they are localised in excretory organs such as the liver, intestine and the blood-brain barrier (BBB) [9], [10], [11], [12]. Two of the most characterized ABC transporters, P-glycoprotein (P-gp, encoded by *MDR1/ABCB1*) and breast cancer resistance protein (Bcrp, encoded by *BCRP/ABCG2*), are both found at the apical surface of capillary endothelial cells that comprise the BBB. P-gp has been shown to regulate the brain accumulation of various central nervous system (CNS) drugs including antipsychotics, selective serotonin reuptake inhibitors, opioids and antiepileptic agents [13], [14], [15]. Similarly Bcrp affects brain disposition of drugs [16] and often cooperates with P-gp to extrude compounds from the brain because these transporters share overlapping substrate specificities [17], [18], [19].

A recent study suggests P-gp regulates the brain disposition of THC, as an *ABCB1* polymorphism was associated with an increased risk of developing cannabis dependence [20]. *In vitro* studies show cannabinoids bind ABC transporters and that THC stimulates P-gp ATPase activity and inhibits Bcrp [21], [22], [23], [24], [25]. Further, CFI mice naturally deficient in P-gp expression accumulate higher plasma levels of THC following oral dosing than wild-type (WT) mice implying that THC is a substrate of P-gp [26]. However no prior studies have directly shown that P-gp and Bcrp regulate the brain disposition of THC.

One strategy to examine whether THC is a substrate of P-gp and Bcrp with implications for its CNS-disposition is to utilize mice devoid of ABC transporter genes [14], [27], [28]. Prior studies have demonstrated that *Abcb1a/b* (−/−) and *Abcg2* (−/−) mice attain higher brain levels of P-gp and Bcrp substrate drugs [14], [19]. Using these mice we aim to show that *Abcb1a/b* (−/−) and *Abcg2* (−/−) retain higher brain levels of THC and that this has functional consequences for CNS-mediated actions of THC.

## Results

### Higher brain and blood levels of THC in *Abcb1a/b* (−/−) and *Abcg2* (−/−) mice

**Figure 1:**
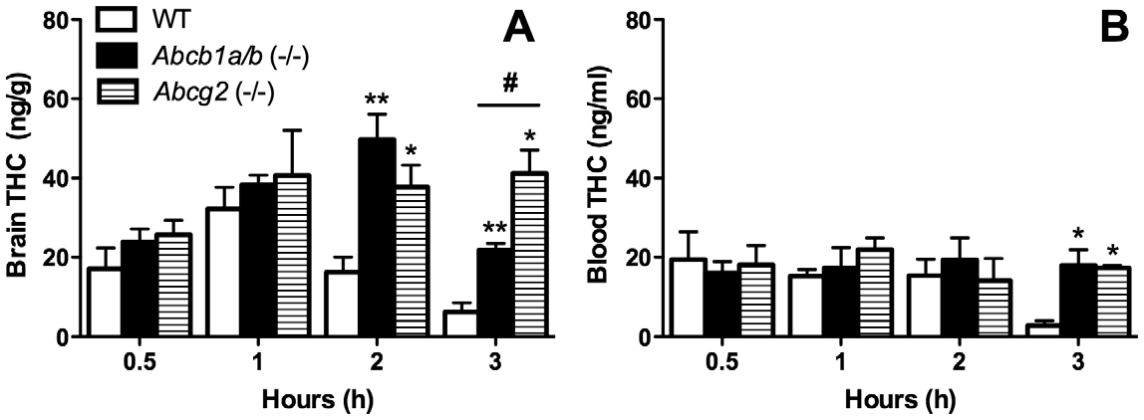
*Abcb1a/b* (−/−) and *Abcg2* (−/−) mice display higher brain and blood THC levels compared to WT mice. Brain (A) and blood (B) THC concentrations in WT, *Abcb1a/b* (−/−) and *Abcg2* (−/−) mice at 0.5, 1, 2 and 3 h after acute administration of THC (mean ± SEM). *Abcb1a/b* (−/−) or *Abcg2* (−/−) compared to WT group at specific time-points * *P*<0.05, ** *P*<0.01, *Abcb1a/b* (−/−) compared to *Abcg2* (−/−), # *P*<0.05. For WT mice n = 6 (0.5 h), n = 5 (1 h), n = 6 (2 h) and n = 5 (3 h). For *Abcb1a/b* (−/−) n = 6 (0.5 h), n = 5 (1 h), n = 6 (2 h) and n = 5 (3 h). For *Abcg2* (−/−) n = 6 (0.5 h), n = 5 (1 h), n = 5 (2 h) and n = 4 (3 h).

Results from brain and blood THC analyses obtained from WT, *Abcb1a/b* (−/−) and *Abcg2* (−/−) mice are represented in Figure 1. Both *Abcb1a/b* (−/−) and *Abcg2* (−/−) mice achieved higher brain concentrations of THC than WT mice when collapsing all time-points together (Figure 1A) [Mann-Whitney U test, *P* = 0.0006; *P* = 0.0002 respectively]. The differences in brain concentrations of THC were evident post-1 h of administration with *Abcb1a/b* (−/−) and *Abcg2* (−/−) mice showing higher brain THC levels than WT at both 2 h (Mann-Whitney U test, *P* = 0.0065 and *P* = 0.0176 respectively) and 3 h (Mann-Whitney U test, *P* = 0.0090 and *P* = 0.0143 respectively). *Abcg2* (−/−) mice also had significantly higher brain THC concentrations than *Abcb1a/b* (−/−) mice 3 hours post-administration (Mann-Whitney U test, *P* = 0.0143). When including all time-points together in the analysis neither *Abcb1a/b* (−/−) or *Abcg2* (−/−) mice showed higher blood THC concentrations than WT (Figure 1B). However, both the *Abcb1a/b* (−/−) and *Abcg2* (−/−) mice (Mann-Whitney U test *P* = 0.0139; *P* = 0.0143) had significantly higher blood THC levels than WT at 3 h.

### Brain/blood THC ratios in *Abcb1a/b* (−/−) and *Abcg2* (−/−) mice

Brain/blood THC concentration ratios for WT, *Abcb1a/b* (−/−) and *Abcg2* (−/−) mice are shown in Table 1. When including all time-points neither *Abcb1a/b* (−/−) or *Abcg2* (−/−) mice displayed higher brain/blood THC concentration ratios compared to WT mice (Mann-Whitney U test, *Ps*>0.05). While not reaching statistical significance, at the 2 h time-point WT mice retained only 1.3 times higher brain THC than blood THC concentrations, whereas *Abcb1a/b* (−/−) and *Abcg2* (−/−) mice attained 4.18 and 6.09 times higher brain THC than blood THC levels respectively (Mann-Whitney U test, *Ps*>0.05).

**Table 1:** Brain/blood THC ratios in *Abcb1a/b* (−/−) and *Abcg2* (−/−) mice compared to WT mice.

	Brain/blood THC ratios			
Time (h)	0.5	1	2	3
WT	1.2±0.52	2.11±0.24	1.30±0.35	1.67±0.44
*Abcb1a/b* (−/−)	1.80±0.37	3.20±0.93	4.18±1.41	1.54±0.45
*Abcg2* (−/−)	3.33±1.78	1.93±0.50	6.09±1.90	2.40±0.35

The ratio of brain to blood THC concentrations in WT, *Abcb1a/b* (−/−) and *Abcg2* (−/−) mice from data collected at 0.5, 1, 2 and 3 h after an acute administration of THC (mean ± SEM).

### Augmented THC-induced hypothermia in *Abcb1a/b* (−/−) and *Abcg2* (−/−) mice

**Figure 2:**
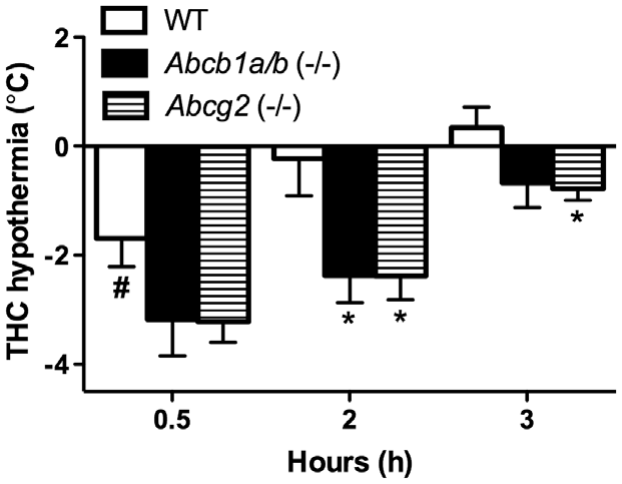
Greater THC-induced hypothermia in *Abcb1a/b* (−/−) and *Abcg2* (−/−) mice compared to WT mice. THC-induced hypothermia in WT, *Abcb1a/b* (−/−) and *Abcg2* (−/−) mice at 0.5, 2 and 3 h post-THC injection. Data expressed as a change from baseline (pre-THC injection) rectal body temperature reading in these mice at the various time-points (mean ± SEM). *Abcb1a/b* (−/−) or *Abcg2* (−/−) compared to WT group at specific time-points * P<0.05, baseline versus post-THC injection body temperature in WT mice # *P*<0.05. For WT mice n = 7, *Abcb1a/b* (−/−) n = 6 and *Abcg2* (−/−) n = 6.

Given that both *Abcb1a/b* (−/−) and *Abcg2* (−/−) mice retained higher concentrations of THC in the brain we proceeded to test whether this had functional consequences by examining whether *Abcb1a/b* (−/−) and *Abcg2* (−/−) mice were more sensitive than WT mice to the hypothermic actions of THC - an effect mediated by the CNS (see Figure 2). THC exposure in WT mice significantly reduced body temperature from baseline at 30 min post-injection (Wilcoxon test, *P* = 0.0180) and this hypothermia disappeared 2–3 h following THC exposure. *Abcg2* (−/−) mice had significantly greater THC-induced hypothermia than WT mice at the 2 and 3 h time-points post-injection (Mann-Whitney U test, *P* = 0.0452 and *P* = 0.0319 respectively). *Abcb1a/b* (−/−) mice had significantly greater hypothermia than WT mice at 2 h post-injection only (Mann-Whitney U test, *P* = 0.0266).

## Discussion

Here we show for the first time that the ABC transporters P-gp and Bcrp regulate the brain disposition and consequent CNS-mediated functional effect of the main psychoactive constituent of cannabis, THC. Both *Abcb1a/b* (−/−) and *Abcg2* (−/−) mice retained greater brain THC concentrations and were more sensitive to hypothermia promoted by THC than WT mice. This appeared to be explained by impaired elimination of the drug from the brain, as WT mice showed decreased brain and blood concentrations over time, whereas *Abcb1a/b* (−/−) and *Abcg2* (−/−) mice maintained higher THC concentrations that were first evident in the brain at 2 h post-injection before it was observed in the blood at 3 h. The time-frame of enhanced brain THC concentrations in *Abcb1a/b* (−/−) and *Abcg2* (−/−) mice mirrored that observed for THC-induced hypothermia – an effect mediated by the CNS.

Our results accord with prior *in vitro* and *in vivo* evidence indicating THC is a substrate of P-gp. THC was first shown to have affinity for P-gp as it weakly stimulated human P-gp-dependent ATPase activity [25]. Bonhomme-Faivre et al. (2008) then demonstrated THC is a P-gp substrate by showing that CFI mice, which are naturally deficient in *Abcb1a*, attain higher plasma levels of THC than WT mice following a 25 mg/kg oral THC dose [26]. Our results extend this research by showing P-gp regulates brain disposition of THC. Further, we provide first evidence that THC is a substrate of Bcrp and that this transporter regulates brain and blood levels of THC. This finding is consistent with our prior *in vitro* evidence showing THC inhibits Bcrp [22] and suggests THC-induced enhancement of mitoxantrone accumulation in Bcrp-overexpressing cells occurs via competitive modulation of Bcrp transport.

Our results highlight that THC is a dual substrate for P-gp and Bcrp which is not surprising given the considerable overlap in the substrate specificities of P-gp and Bcrp and that many drugs are transported by both these efflux proteins [17], [18], [19], [32]. THC appears a better substrate for Bcrp than P-gp because *Abcg2* (−/−) mice retained higher levels of THC than *Abcb1a/b* (−/−) mice in the brain at 3 h post-administration. This is at odds with findings that P-gp has approximately 5-fold higher expression in murine brain vascular endothelial cells than Bcrp [33], [34]. However, our laboratory has shown that THC inhibits Bcrp but not P-gp because THC increases accumulation of the Bcrp substrate mitoxantrone but not the P-gp substrate rhodamine 123 in cells selectively expressing these respective transporters [22], [23]. Taken together, our results imply THC may have significantly lower affinity for P-gp than Bcrp.

Our results suggest that P-gp and Bcrp expressed on endothelial cells that comprise the BBB assist in the active transport of THC from inside the brain back into the peripheral blood supply. *Abcb1a/b* (−/−) and *Abcg2* (−/−) mice, without these transporters, display delayed brain elimination of THC leading to the accumulation of this psychoactive constituent of cannabis in the CNS. This implies that *Abcb1a/b* (−/−) and *Abcg2* (−/−) mice could also be more sensitive to CNS-mediated functional effects of THC. It is well established that THC promotes hypothermia through its action on the CNS largely by affecting thermoregulatory neurons in the hypothalamus [35]. For example, a classic study by Fitton and Pertwee (1982) demonstrated that intracerebroventricular and intrahypothalamic injections of THC promoted reductions in rectal body temperature in mice [36]. Here we show that mice devoid of P-gp or Brcp are more sensitive to hypothermia promoted by THC – a likely functional consequence of enhanced THC concentrations in the brain affecting thermoregulatory neurons in these mice.

Our results offer an additional pharmacokinetic mechanism for interactions between the approximately 80 different cannabinoid constituents found in the cannabis plant. Interaction between THC and another plant-derived cannabinoid constituent cannabidiol (CBD) is of particular interest as CBD modulates the actions of THC [31], [37], [38], [39], [40], [41]. Indeed this was part of the rationale behind the development of Sativex®, a cannabinoid formulation containing a 1.07∶1 THC ∶ CBD dose, approved for use in various countries for the treatment of spasticity associated with multiple sclerosis. CBD's modulation of THC effects might be partly explained by pharmacokinetic mechanisms because CBD increases the brain levels of THC and accentuates some of its neurobehavioural effects [31], [37], [42]. Our present findings showing THC is a substrate of P-gp and Bcrp, combined with prior research indicating CBD inhibits both P-gp and Bcrp [23], [25], highlights a novel ABC transporter-mediated mechanism whereby CBD might inhibit the transport of THC from the brain parenchyma back into the blood.

Cannabis-drug interactions should also be considered in future research as substrates for P-gp and Bcrp include several CNS drugs that might be co-administered with cannabis such as anticonvulsants, antidepressants and antipsychotics [14], [43], [44], [45], [46], [47], [48], [49]. This may be particularly problematic for antipsychotic treatment as there are higher rates of cannabis use in schizophrenia patients than the general population and cannabis use is associated with poor antipsychotic treatment outcomes [50], [51], [52]. THC behaving as a competitive substrate for P-gp might increase the brain and blood concentrations of P-gp substrate antipsychotic drugs such as risperidone and olanzapine [14], [48], [53] and increase dose-related side-effects of these drugs. Alternatively, antipsychotic drugs by inhibiting P-gp and Bcrp [54] might increase CNS accumulation of THC and increase psychotic reactions and relapse in these patients.

The present findings raise the question of whether *ABCB1* and *ABCG2* polymorphisms might explain genetic susceptibility to cannabis-induced psychosis or the adverse psychoactive effects of this drug (e.g. addiction, anxiety and cognitive impairment) by altering the brain disposition of THC. This would provide a pharmacokinetic source of interindividual variability in response to cannabis use in addition to other genes that modulate the pharmacodynamic actions of the drug [2], [4], [5], [6]. Thus far, to the best of our knowledge, only one human study has addressed whether single nucleotide polymorphisms (SNPs) in ABC transporter genes confers greater vulnerability to CNS disorders. This study showed that the *ABCB1* variant C3435T (CC genotype) increased the risk of developing cannabis dependence, as this SNP was more prevalent in individuals dependent on cannabis than controls [20]. The CC genotype has been associated with increased expression of P-gp, whereas the TT genotype decreased P-gp expression [55]. Therefore, dependent on genotype the SNP C3435T might decrease or increase THC brain levels and on one hand increase the propensity of individuals for cannabis dependence (CC genotype) or increase the risk of developing cannabis-induced psychosis (TT genotype). Our findings reinforce the need for future studies to more closely examine the relationship between SNPs in *ABCB1* and *ABCG2* and cannabis-related psychiatric problems such as addiction, schizophrenia and panic disorder.

In conclusion our results show that the main psychoactive constituent of cannabis THC is a substrate for the ABC transporters P-gp and Bcrp. *Abcb1a/b (−/−)* and *Abcg2 (−/−)* mice attained higher brain THC concentrations and were more sensitive to the CNS-mediated hypothermic effects of THC than WT mice. These results provide a novel mechanism for genetic vulnerability to the psychoactive effects of cannabis, the most widely used illicit drug in the world. Further they highlight a novel mechanism for drug interactions between cannabis use and CNS drugs that are substrates for the ABC transporters P-gp and Bcrp.

## Materials and Methods

### Animals

All Experiments were approved by the University of Sydney Animal Ethics Committee in accordance with the *Australian Code of Practice for the Care and Use of Animals for Scientific Purposes*. Age-matched female *Abcb1a/b* (−/−), *Abcg2* (−/−) and wild-type (WT, FVB background strain) mice were used in this study. *Abcb1a/b* (−/−) and *Abcg2* (−/−) mice were purchased from Taconic farms (New York, USA) and were developed by Professor Alfred Schinkel and colleagues at the Netherlands Cancer Institute, Amsterdam (see [27], [28] for the initial characterisation of these animals). Female mice were used to allow comparison of our data with a prior study that assessed the disposition of numerous CNS drugs in female WT and *Abcb1a/b* (−/−) mice [14]. Following shipment of founder mice to Australia from the US all mice genotypes were bred in-house and the offspring were used for experimentation following rearing under identical conditions. Mice were 2–3 months old for our first study assessing the brain and blood concentrations of THC and 4.5 months old for the second study examining THC-induced hypothermia. All mice were group-housed with 4–6 mice per cage in a temperature and humidity controlled room and maintained on a reverse 12-h light/12-h dark cycle. Experimental sessions took place during the dark cycle. Mice had *ad libitum* access to water and rodent lab chow.

### Drug treatment

Δ^9^-Tetrahydrocannabinol (THC Pharm GmbH, Frankfurt) was dissolved in absolute ethanol before being added to an equal amount of Tween 80 and diluted in 0.9% saline to give a final stock of ethanol/Tween 80/saline (1∶1∶18). All drugs were freshly prepared before use at an injection volume of 1 ml/kg. THC was administered intraperitoneally (i.p.) at a dose of 3 mg/kg for the measurement of THC concentration in brain and blood. At 0.5, 1, 2 and 3 h post-THC injections all mice (*Abcb1a/b* (−/−), *Abcg2* (−/−) and WT animals, n = 5–6 per group) were anaesthetised with isoflurane and blood was collected via cardiac puncture before brains were extracted and snap frozen in liquid nitrogen. To avoid coagulation blood was stored in EDTA tubes. Both brain and blood were stored at −20°C prior to gas chromatography mass spectrometry (GC-MS) analysis. For the body temperature study mice (n = 6–7 per group) were first tested for baseline rectal body temperature as previously described (Boucher *et al*, 2011b). All mice were then administered an i.p. injection of 10 mg/kg THC before rectal body temperature measurements at 30 min, 2 h and 3 h post-injection were taken. The higher 10 mg/kg dose of THC was administered in this study as, unlike 3 mg/kg THC, this dose significantly reduced body temperature in WT mice, allowing observation of any potentiation of this effect in *Abcb1a/b* (−/−) and *Abcg2* (−/−) mice.

### GC-MS analysis of THC in biological matrices

The methods used for determining brain and blood THC concentrations were as described previously [29], [30], [31]. Briefly for blood THC analysis, a total of 50 µl of (deuterated) D3-THC (0.25 mg/L) internal standard solution was added to each 0.5 ml sample of whole blood. Acetate buffer was added (pH 4.0) to THC and was extracted with 1-chlorobutane solvent. Calibration standards were prepared by spiking drug-free sheep blood at concentrations of 1.25–100 ng/ml for THC. The standards were vortexed and treated identically to other blood samples. Following extraction and complete drying under a nitrogen gas stream, the samples underwent derivatization of the polar functional groups (COOH, OH) with bis(trimethylsilyl)trifluroacetamide (BSTFA). The limit of quantification (LOQ) for the blood analysis was 1.25 ng/ml. For brain THC analysis, brains were dissected in half in the sagittal plane and each hemisphere was weighed. A single hemisphere was then homogenized in 0.9% saline at a 1∶2 ratio (w/v). A total of 50 µl of (deuterated) D3-THC (0.25 mg/L) internal standard solution, 2 ml of 0.2 M sodium hydroxide (pH 13) and 3 ml of acetonitrile were added to each 0.5 ml sample of homogenized brain samples. Calibration standards were prepared by spiking drug-free mouse brains at concentrations of 5–200 ng/g for THC. They were then completely dried under nitrogen gas stream and underwent identical extraction and derivatization as blood samples, as described above (LOQ = 5 ng/g for the brain analysis). All quantification was performed by GC-MS (Shimadzu 2010Plus system) using electron impact ionization in selective ion mode.

### Data analysis

All statistical analyses were performed using StatView 5.01 (SAS Institute Inc. Cary, NC, USA). Comparisons were made between *Abcb1a/b* (−/−) or *Abcg2* (−/−) mice versus WT from time-point data collapsed together or analysed separately using the non-parametric Mann-Whitney U test. A Wilcoxon test was used to demonstrate that THC promoted significant hypothermia in WT mice by comparing the various time-points to baseline (the pre-THC injection body temperature). Differences were deemed significant when *P*<0.05.

## References

[1] Henquet C, Di Forti M, Morrison P, Kuepper R, Murray RM (2008). Gene-environment interplay between cannabis and psychosis.. Schizophr Bull.

[2] Caspi A, Moffitt TE, Cannon M, McClay J, Murray R (2005). Moderation of the effect of adolescent-onset cannabis use on adult psychosis by a functional polymorphism in the catechol-O-methyltransferase gene: longitudinal evidence of a gene X environment interaction.. Biol Psychiatry.

[3] O'Tuathaigh CM, Hryniewiecka M, Behan A, Tighe O, Coughlan C Chronic adolescent exposure to Delta-9-tetrahydrocannabinol in COMT mutant mice: impact on psychosis-related and other phenotypes.. Neuropsychopharmacology.

[4] Boucher AA, Arnold JC, Duffy L, Schofield PR, Micheau J (2007a). Heterozygous neuregulin 1 mice are more sensitive to the behavioural effects of Delta9-tetrahydrocannabinol.. Psychopharmacology (Berl).

[5] Boucher AA, Hunt GE, Karl T, Micheau J, McGregor IS (2007b). Heterozygous neuregulin 1 mice display greater baseline and Delta(9)-tetrahydrocannabinol-induced c-Fos expression.. Neuroscience.

[6] Boucher AA, Hunt GE, Micheau J, Huang X, McGregor IS (2011b). The schizophrenia susceptibility gene neuregulin 1 modulates tolerance to the effects of cannabinoids.. Int J Neuropsychopharmacol.

[7] Long LE, Chesworth R, Huang XF, McGregor IS, Arnold JC (2011). A behavioural comparison of acute and chronic Delta9-tetrahydrocannabinol and cannabidiol in C57BL/6JArc mice.. Int J Neuropsychopharmacol.

[8] Hall MD, Handley MD, Gottesman MM (2009). Is resistance useless? Multidrug resistance and collateral sensitivity.. Trends Pharmacol Sci.

[9] Cordon-Cardo C, O'Brien JP, Boccia J, Casals D, Bertino JR (1990). Expression of the multidrug resistance gene product (P-glycoprotein) in human normal and tumor tissues.. J Histochem Cytochem.

[10] Langmann T, Mauerer R, Zahn A, Moehle C, Probst M (2003). Real-time reverse transcription-PCR expression profiling of the complete human ATP-binding cassette transporter superfamily in various tissues.. Clin Chem.

[11] Schinkel AH (1997). The physiological function of drug-transporting P-glycoproteins.. Semin Cancer Biol.

[12] Zimmermann C, Gutmann H, Hruz P, Gutzwiller JP, Beglinger C (2005). Mapping of multidrug resistance gene 1 and multidrug resistance-associated protein isoform 1 to 5 mRNA expression along the human intestinal tract.. Drug Metab Dispos.

[13] Baltes S, Gastens AM, Fedrowitz M, Potschka H, Kaever V (2007). Differences in the transport of the antiepileptic drugs phenytoin, levetiracetam and carbamazepine by human and mouse P-glycoprotein.. Neuropharmacology.

[14] Doran A, Obach RS, Smith BJ, Hosea NA, Becker S (2005). The impact of P-glycoprotein on the disposition of drugs targeted for indications of the central nervous system: evaluation using the MDR1A/1B knockout mouse model.. Drug Metab Dispos.

[15] Tournier N, Decleves X, Saubamea B, Scherrmann JM, Cisternino S (2011). Opioid transport by ATP-Binding Cassette (ABC) Transporters at the Blood-Brain Barrier: Implications for Neuropsychopharmacology.. Curr Pharm Des.

[16] Urquhart BL, Kim RB (2009). Blood-brain barrier transporters and response to CNS-active drugs.. Eur J Clin Pharmacol.

[17] de Vries NA, Zhao J, Kroon E, Buckle T, Beijnen JH (2007). P-glycoprotein and breast cancer resistance protein: two dominant transporters working together in limiting the brain penetration of topotecan.. Clin Cancer Res.

[18] Yang JJ, Milton MN, Yu S, Liao M, Liu N (2010). P-glycoprotein and breast cancer resistance protein affect disposition of tandutinib, a tyrosine kinase inhibitor.. Drug Metab Lett.

[19] Lagas JS, van Waterschoot RA, van Tilburg VA, Hillebrand MJ, Lankheet N (2009). Brain accumulation of dasatinib is restricted by P-glycoprotein (ABCB1) and breast cancer resistance protein (ABCG2) and can be enhanced by elacridar treatment.. Clin Cancer Res.

[20] Benyamina A, Bonhomme-Faivre L, Picard V, Sabbagh A, Richard D (2009). Association between ABCB1 C3435T polymorphism and increased risk of cannabis dependence.. Prog Neuropsychopharmacol Biol Psychiatry.

[21] Holland ML, Allen JD, Arnold JC (2008). Interaction of plant cannabinoids with the multidrug transporter ABCC1 (MRP1).. Eur J Pharmacol.

[22] Holland ML, Lau DT, Allen JD, Arnold JC (2007). The multidrug transporter ABCG2 (BCRP) is inhibited by plant-derived cannabinoids.. Br J Pharmacol.

[23] Holland ML, Panetta JA, Hoskins JM, Bebawy M, Roufogalis BD (2006). The effects of cannabinoids on P-glycoprotein transport and expression in multidrug resistant cells.. Biochem Pharmacol.

[24] Nieri P, Romiti N, Adinolfi B, Chicca A, Massarelli I (2006). Modulation of P-glycoprotein activity by cannabinoid molecules in HK-2 renal cells.. Br J Pharmacol.

[25] Zhu HJ, Wang JS, Markowitz JS, Donovan JL, Gibson BB (2006). Characterization of P-glycoprotein inhibition by major cannabinoids from marijuana.. J Pharmacol Exp Ther.

[26] Bonhomme-Faivre L, Benyamina A, Reynaud M, Farinotti R, Abbara C (2008). Disposition of Delta tetrahydrocannabinol in CF1 mice deficient in mdr1a P-glycoprotein.. Addict Biol.

[27] Jonker JW, Buitelaar M, Wagenaar E, Van Der Valk MA, Scheffer GL (2002). The breast cancer resistance protein protects against a major chlorophyll-derived dietary phototoxin and protoporphyria.. Proc Natl Acad Sci U S A.

[28] Schinkel AH, Mayer U, Wagenaar E, Mol CA, van Deemter L (1997). Normal viability and altered pharmacokinetics in mice lacking mdr1-type (drug-transporting) P-glycoproteins.. Proc Natl Acad Sci U S A.

[29] Gunasekaran N, Long LE, Dawson BL, Hansen GH, Richardson DP (2009). Reintoxication: the release of fat-stored delta(9)-tetrahydrocannabinol (THC) into blood is enhanced by food deprivation or ACTH exposure.. Br J Pharmacol.

[30] Quinn HR, Matsumoto I, Callaghan PD, Long LE, Arnold JC (2008). Adolescent rats find repeated Delta(9)-THC less aversive than adult rats but display greater residual cognitive deficits and changes in hippocampal protein expression following exposure.. Neuropsychopharmacology.

[31] Klein C, Karanges E, Spiro A, Wong A, Spencer J (2011). Cannabidiol potentiates Delta(9)-tetrahydrocannabinol (THC) behavioural effects and alters THC pharmacokinetics during acute and chronic treatment in adolescent rats.. Psychopharmacology (Berl).

[32] Breedveld P, Beijnen JH, Schellens JH (2006). Use of P-glycoprotein and BCRP inhibitors to improve oral bioavailability and CNS penetration of anticancer drugs.. Trends Pharmacol Sci.

[33] Warren MS, Zerangue N, Woodford K, Roberts LM, Tate EH (2009). Comparative gene expression profiles of ABC transporters in brain microvessel endothelial cells and brain in five species including human.. Pharmacol Res.

[34] Kamiie J, Ohtsuki S, Iwase R, Ohmine K, Katsukura Y (2008). Quantitative atlas of membrane transporter proteins: development and application of a highly sensitive simultaneous LC/MS/MS method combined with novel in-silico peptide selection criteria.. Pharm Res.

[35] Wenger T, Moldrich G (2002). The role of endocannabinoids in the hypothalamic regulation of visceral function.. Prostaglandins Leukot Essent Fatty Acids.

[36] Fitton AG, Pertwee RG (1982). Changes in body temperature and oxygen consumption rate of conscious mice produced by intrahypothalamic and intracerebroventricular injections of delta 9-tetrahydrocannabinol.. Br J Pharmacol.

[37] Bornheim LM, Kim KY, Li J, Perotti BY, Benet LZ (1995). Effect of cannabidiol pretreatment on the kinetics of tetrahydrocannabinol metabolites in mouse brain.. Drug Metab Dispos.

[38] Malone DT, Jongejan D, Taylor DA (2009). Cannabidiol reverses the reduction in social interaction produced by low dose Delta(9)-tetrahydrocannabinol in rats.. Pharmacol Biochem Behav.

[39] Morgan CJ, Curran HV (2008). Effects of cannabidiol on schizophrenia-like symptoms in people who use cannabis.. Br J Psychiatry.

[40] Morgan CJ, Schafer G, Freeman TP, Curran HV (2010). Impact of cannabidiol on the acute memory and psychotomimetic effects of smoked cannabis: naturalistic study: naturalistic study [corrected].. Br J Psychiatry.

[41] Murphy LL, Steger RW, Smith MS, Bartke A (1990). Effects of delta-9-tetrahydrocannabinol, cannabinol and cannabidiol, alone and in combinations, on luteinizing hormone and prolactin release and on hypothalamic neurotransmitters in the male rat.. Neuroendocrinology.

[42] Varvel SA, Wiley JL, Yang R, Bridgen DT, Long K (2006). Interactions between THC and cannabidiol in mouse models of cannabinoid activity.. Psychopharmacology (Berl).

[43] Hemauer SJ, Patrikeeva SL, Wang X, Abdelrahman DR, Hankins GD Role of transporter-mediated efflux in the placental biodisposition of bupropion and its metabolite, OH-bupropion.. Biochem Pharmacol.

[44] Lazarowski A, Czornyj L, Lubienieki F, Girardi E, Vazquez S (2007). ABC transporters during epilepsy and mechanisms underlying multidrug resistance in refractory epilepsy.. Epilepsia.

[45] O'Brien FE, Dinan TG, Griffin BT, Cryan JF Interactions between antidepressants and P-glycoprotein at the blood-brain barrier: Clinical significance of in vitro and in vivo findings.. Br J Pharmacol.

[46] Potschka H Modulating P-glycoprotein regulation: future perspectives for pharmacoresistant epilepsies?. Epilepsia.

[47] Schinkel AH, Jonker JW (2003). Mammalian drug efflux transporters of the ATP binding cassette (ABC) family: an overview.. Adv Drug Deliv Rev.

[48] Wang JS, Taylor R, Ruan Y, Donovan JL, Markowitz JS (2004). Olanzapine penetration into brain is greater in transgenic Abcb1a P-glycoprotein-deficient mice than FVB1 (wild-type) animals.. Neuropsychopharmacology.

[49] Yanase K, Tsukahara S, Mitsuhashi J, Sugimoto Y (2006). Functional SNPs of the breast cancer resistance protein-therapeutic effects and inhibitor development.. Cancer Lett.

[50] Bowers MB, Mazure CM, Nelson JC, Jatlow PI (1990). Psychotogenic drug use and neuroleptic response.. Schizophr Bull.

[51] Hall W, Degenhardt L, Teesson M (2004). Cannabis use and psychotic disorders: an update.. Drug Alcohol Rev.

[52] Turner WM, Tsuang MT (1990). Impact of substance abuse on the course and outcome of schizophrenia.. Schizophr Bull.

[53] Wang JS, Ruan Y, Taylor RM, Donovan JL, Markowitz JS (2004). The brain entry of risperidone and 9-hydroxyrisperidone is greatly limited by P-glycoprotein.. Int J Neuropsychopharmacol.

[54] Wang JS, Zhu HJ, Markowitz JS, Donovan JL, Yuan HJ (2008). Antipsychotic drugs inhibit the function of breast cancer resistance protein.. Basic Clin Pharmacol Toxicol.

[55] Hoffmeyer S, Burk O, von Richter O, Arnold HP, Brockmoller J (2000). Functional polymorphisms of the human multidrug-resistance gene: multiple sequence variations and correlation of one allele with P-glycoprotein expression and activity in vivo.. Proc Natl Acad Sci U S A.

